# PACAP and its receptors in cranial arteries and mast cells

**DOI:** 10.1186/s10194-017-0822-2

**Published:** 2018-02-20

**Authors:** Inger Jansen-Olesen, Sara Hougaard Pedersen

**Affiliations:** 1 Danish Headache Center, Department of Neurology, Glostrup Research Institute, Rigshospitalet and Faculty of Health and Medical Sciences, University of Copenhagen, Copenhagen, Denmark; 2 Department of Neurology, Danish Headache Center, Glostrup Research Institute, Nordre Ringvej 69, 2600 Glostrup, Denmark

**Keywords:** Migraine, PACAP, VIP, Cerebral artery, Middle meningeal artery, Mast cells

## Abstract

**Background:**

In migraineurs pituitary adenylate cyclase activating peptide1–38 (PACAP1–38) is a potent migraine provoking substance and the accompanying long lasting flushing suggests degranulation of mast cells. Infusion of the closely related vasoactive intestinal peptide (VIP) either induces headache or flushing. This implicates the pituitary adenylate cyclase activating peptide type I receptor (PAC1) to be involved in the pathophysiology of PACAP1–38 provoked headaches. Here we review studies characterizing the effects of mainly PACAP but also of VIP on cerebral and meningeal arteries and mast cells.

**Discussion:**

PACAP1–38, PACAP1–27 and VIP dilate cerebral and meningeal arteries from several species including man. In rat cerebral and meningeal arteries the dilation seems to be mediated preferably via vasoactive intestinal peptide receptor type 1 (VPAC1) receptors while, in human, middle meningeal artery dilation induced via vasoactive intestinal peptide receptor type 2 (VPAC2) receptors cannot be ruled out. PACAP1–38 is a strong degranulator of peritoneal and dural mast cells while PACAP1–27 and VIP only have weak effects. More detailed characterization studies suggest that mast cell degranulation is not mediated via the known receptors for PACAP1–38 but rather via a still unknown receptor coupled to phospholipase C.

**Conclusion:**

It is suggested that PACAP1–38 might induce migraine via degranulation of dural mast cells via a yet unknown receptor.

## Review

Migraine is number six in WHO list of all diseases causing disability [1] and it is the third most costly neurological disorder in Europe [2]. Even though the triptans revolutionized the acute treatment of migraine, a huge unmet need for better or different acute treatments exists [3]. An interesting molecule in this aspect is pituitary adenylate cyclase activating peptide (PACAP), which exists in the body as 38- and 27- amino acid peptides [4, 5]. These peptides partly share receptors with their family member vasoactive intestinal peptide (VIP) [6]. In migraineurs, elevated levels of PACAP1–38 were found in blood sampled from the external jugular vein [7] and cubital vein [8] during migraine attacks. Infusion of PACAP1–38 provokes immediate headache in 11 out of 12 migraine patients, 7 of these patients develop delayed migraine attacks. In all 12 healthy subjects an immediate headache was experienced, two of these subsequently reporting migraine-like symptoms [9, 10]. Interestingly, VIP only induces a mild headache and no migraine-like attacks in migraineurs [11]. These findings point towards the PAC1 receptor, which is targeted by PACAP with much higher affinity than VIP, as a key target for migraine treatment. In this review we describe studies characterizing the receptors upon which PACAP and VIP mediate dilation of intracranial arteries and degranulation of peritoneal and dural mast cells.

### Pituitary adenylate cyclase activating peptide

Pituitary adenylate cyclase activating peptide (PACAP) is a highly conserved signaling peptide of identical structure in mammals including human, sheep, rat and mouse [12]. It is a member of the glucagon/secretin superfamily of peptides [6, 13, 14] and is endogenously present in two isoforms namely; PACAP1–38 and the C-terminal truncated version PACAP1–27. High concentrations of PACAP1–38 are found in brain and in testis. Especially the hypothalamus but also other brain regions contain considerable amounts of PACAP1–38. PACAP1–27 is considerably less abundant in these regions as compared to PACAP1–38 [4]. A related member of the glucagon/secretin superfamily is the 28 amino acid peptide, VIP that shares 68% homology with PACAP1–27 from the N-terminal end. PACAP and VIP are signaling molecules widely distributed throughout the central and peripheral nervous system [6, 13] involved in e.g. regulation of circadian rhythm [15], neuroprotection [16, 17], inflammation and pain perception [18, 19].

PACAP-immunoreactivity (−IR) and VIP-IR co-localize in nerve fibers innervating cerebral vessels and parasympathetic ganglia [20–24] and in dura mater where it occasionally co-localizes with calcitonin gene-related peptide (CGRP) [25]. In trigeminal ganglion, PACAP-IR co-localizes with CGRP-IR neurons, while only PACAP-IR is found in satellite glial cells [26–28]. In the spinal trigeminal nucleus PACAP-IR co-localizes with CGRP-IR in nerve fibers in laminae I and II [26, 29].

### PACAP receptors

**Fig. 1:**
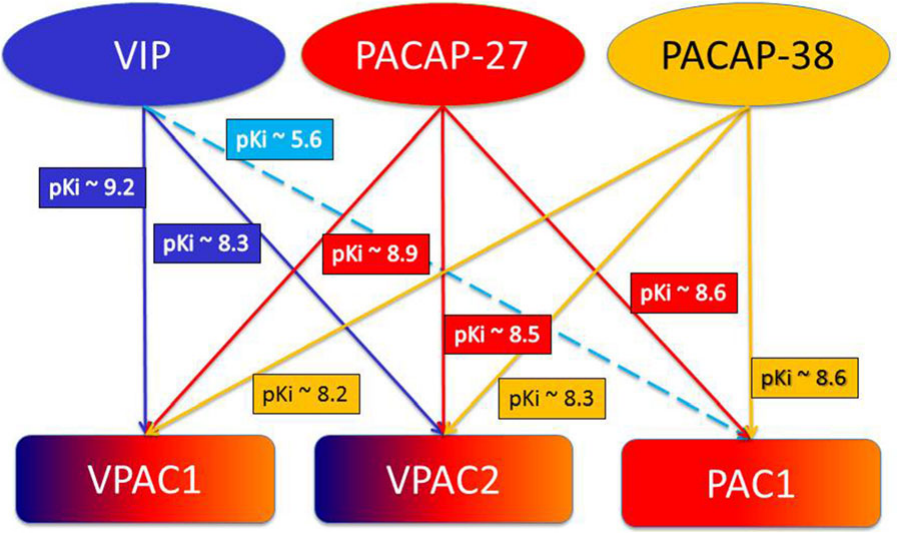
Schematic overview of selectivity of receptors for pituitary adenylate cyclase activating polypeptide (PACAP) and vasoactive intestinal peptide (VIP). Pituitary adenylate cyclase activating polypeptide receptor 1 (PAC1) has a 1000-fold greater affinity for PACAP1–27 (red) and PACAP1–38 (yellow) than for VIP (light blue). Vasoactive intestinal peptide receptor (VPAC)1 and VPAC2 bind VIP (blue) and PACAP1–27(red) and PACAP1–38 (yellow) with equal affinity. pKi (negative logarithm of the concentration that occupies half the receptor population at equilibrium) values given in the figure is adapted from [19]. No difference in receptor selectivity between PACAP1–38 and PACAP1–27 is described

PACAP and VIP partially share receptors and PACAP signal transduction is mediated through three high-affinity G protein-coupled receptors namely pituitary adenylate cyclase activating peptide type I receptor (PAC1), vasoactive intestinal peptide receptor type 1 (VPAC1), and vasoactive intestinal peptide receptor type 2 (VPAC2). The affinities of PACAP1–38 and PACAP1–27 are equal to that of VIP for VPAC1- and VPAC2- receptors, whereas the affinity of PACAP1–38 and PACAP1–27 for the PAC1 receptor (PAC1-R) is about 1000-fold higher than that of VIP [6, 19, 30] (Fig. 1). The potent headache provoking property of PACAP1–38 [10] in comparison with the poor effect of VIP [11], suggests PAC1- R to be an interesting target for migraine treatment.

In human cerebral and middle meningeal arteries, messenger RNA (mRNA) for VPAC1, VPAC2 and PAC1 receptors has been identified [31, 32]. In rat, mRNA of the same three receptors was shown by qPCR in middle meningeal arteries [33] and by in situ hybridization to be localized in smooth muscle cells of middle cerebral arteries, basilar arteries and middle meningeal arteries [34]. Immunohistochemistry with antibodies for the VPAC1 receptor shows its presence in the smooth muscle cells of rat cerebral arteries [24]. In rat trigeminal ganglion and spinal trigeminal nucleus all three receptors are detected at the mRNA level [26, 33].

### Cranial arteries and migraine

In the 1940’s the genesis of migraine pain was attributed to meningeal and cerebral arteries, as it was reported that electrical stimulation of these arteries evoked nausea and ipsilateral pain, localized to the area in and around the eye, including the forehead and temple [35, 36]. The perivascular proximity of nociceptive afferents [37, 38], the pulsating nature of migraine headache (in 80% of patients) aggravating with physical activity [39] as well as pain and nausea induction during arterial stimulation [36], have all been interpreted as strong indicators of a vascular component of migraine pathogenesis. However, accumulating evidence has challenged the theory of migraine as a vascular disease. Migraine-provoking substances are strong vasodilators [10, 40–43]. However, not all vasodilatory compounds provoke accompanying headache [11, 44]. It was recently shown that spontaneous migraine attacks in patients are accompanied by dilation of the pain sensitive middle cerebral and internal carotid arteries whereas no dilation of dural and extracranial arteries are observed [45].

### The effect of PACAP on cerebral arteries

#### In vitro

**Fig. 2:**
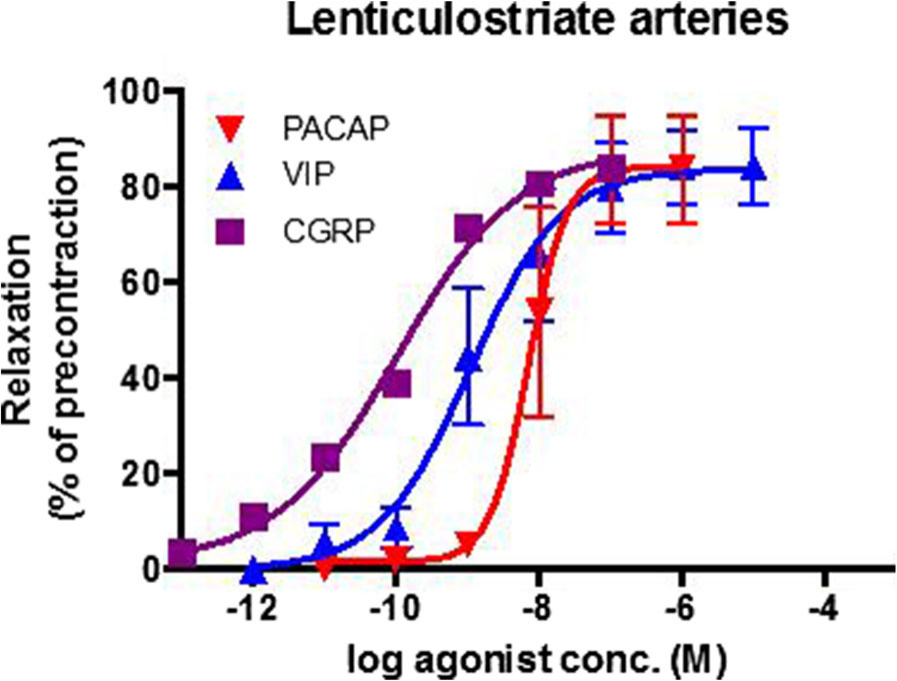
Relaxant responses to PACAP1–27 (*n* = 4), VIP (*n* = 7) and CGRP (*n* = 10), expressed as % of pre-contraction induced by prostaglandin F_2α_ in human cerebral arteries. Mean values ± S.E.M. are given. *n* = number of experiments, one from each patient. Modified from Jansen-Olesen et al. [48]

The relaxant effect of PACAP has been studied on isolated cerebral arteries from several species including humans. The potency of PACAP1–38 and PACAP1–27 given as pD2 values (the negative logarithm to base 10 of the concentration of a drug that gives half-maximal response) are in most specimens around 8 (Table I). In cat the potency and efficacy for VIP were somewhat higher than for PACAP1–38 and PACAP1–27 [46], while no difference in potency was found between PACAP1–27 and VIP in rabbit [23]. PACAP1–27 is less potent as a dilator of human cerebral arteries than calcitonin gene-related peptide (CGRP) and VIP (Fig. 2). Comparing data from two different studies performed in human cerebral arteries, one with PACAP1–38 and the other using PACAP1–27, the relaxations were of the same potency, but PACAP1–38 has a lower efficacy than PACAP1–27 (Table 1) [47, 48]. This observation was also made in rat using pressurized arteriography [47] but not in a wire myography study [34]. However, a direct comparison of PACAP1–38 and PACAP1–27 induced effects on human cerebral arteries in parallel experiments has yet to be performed. Blockade experiments suggest VPAC1 receptors to be of importance for PACAP and VIP induced relaxation of rat middle cerebral and basilar arteries [34].

**Table 1:** Data on relaxant responses induced by PACAP1–38, PACAP1–27 and VIP performed *in vitro* on cerebral arteries from different species

Species	PACAP1–38			PACAP1–27			VIP			Reference
	*pD2*	*Emax*	*n*	*pD2*	*Emax*	*n*	*pD2*	*Emax*	*n*	
Cat	7.9 ± 0.2	24 ± 9	7	7.7 ± 0.4	31 ± 5	7	8.3 ± 0.1	68 ± 8	8	Jansen-Olesen et al. 1994 [46]
Rabbit	n.d.	n.d.	–	8.0 ± 0.1	53 ± 11	6	8.1 ± 0.1	73 ± 5	6	Dalsgaard et al. 2003^a^ [23]
Rat	7.8 ± 0.1	48 ± 7	8	8.0 ± 0.1	51 ± 3	8	8.0 ± 0.2	52 ± 8	7	Baun et al. 2011 [34]
Rat	n.d.	n.d.	–	n.d.	n.d.	–	9.2 ± 0.2	32 ± 1	14	Erdling et al. 2013 [90]
Rat^PA^	7.6 ± 0.2	10 ± 1	5	7.8 ± 0.1	25 ± 1	4	8.7 ± 0.1	28 ± 1	4	Grände et al. 2013 [47]
Human	n.d.	n.d.	–	8.2 ± 0.2	84 ± 9	4	8.8 ± 0.4	84 ± 9	7	Jansen-Olesen et al. 2004 [48]
Human	8.4 ± 0.5	27 ± 8	5	n.d.	n.d.	–	8.1 ± 0.1	57 ± 9	5	Grände et al. 2013 [47]

Data is given as pD2 (negative log concentration inducing half maximum relaxant response), Emax (maximum relaxant response in % of pre-contraction or maximum dilatory capacity), *n* = number of experiments

^PA^Pressurized arteriography abluminal application

^a^PACAP1–27 was used because it induced a stronger response than PACAP1–38

#### In vivo

No studies describe the *in vivo* effect of PACAP on cerebral arteries after i.v. infusion to laboratory animals. The reason for this is most probably due to the fact that PACAP has to cross the blood–brain barrier to reach its receptors in the smooth muscle cells of cerebral arteries. A transport mechanism for PACAP1–38 has been described, which is dependent on the peptide transport system-6 (PTS-6) [49]. However, only a small percentage (0.053%) of PACAP-38 enters the brain after intravenous administration [50]. If a dilation of cerebral arteries is achieved together with a fall in mean arterial blood pressure the interpretation of the results is made complicated due to activation of autoregulatory mechanisms leading to dilation of cerebral arteries [51]. To avoid confusion about dilation of cerebral arteries, pharmacological substances can be infused via an indwelling catheter in the common carotid artery (i.c.), which allows cerebral arteries to be studied without systemic effects [52]. However, no studies has to date been performed to investigate the effect of PACAP1–38 on cerebral arteries after i.c. infusion. In human experimental studies PACAP1–38 infusion in healthy volunteers [53] and migraine patients [54] showed a minor short-lasting dilation of middle cerebral arteries. The measurement of middle cerebral artery diameter in these studies was calculated from blood velocity in the middle cerebral artery and was therefore indirect. In another study, no change in mean circumference of middle cerebral artery was found after infusion of PACAP1–38. Here magnetic resonance angiography was used, which is a more direct way to measure the artery diameter and is superior to measurement of blood velocity [9].

### The effect of PACAP on middle meningeal arteries

#### In vitro

**Fig. 3:**
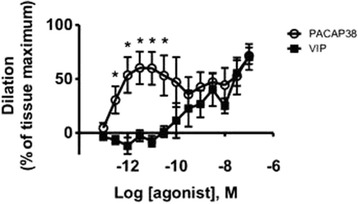
Low picomolar concentrations of PACAP, but not VIP, dilate isolated pressurized rat middle meningeal arteries. Cumulative concentrations of PACAP and VIP were administered to arterial segments pressurized to 40 mmHg *ex vivo*. Arteries were exposed to aCSF containing each concentration of PACAP1–38 or VIP for 20 min. Dilation to PACAP1–38 or VIP are expressed as percentage of maximum dilation obtained in the presence of Ca^2+^ −free artificial CSF containing 100 μM of the calcium channel blocker diltiazem and 1 μM of the adenylyl cyclase activator forskolin. *p* < 0.05 by unpaired t test, *n* = 4. From Syed et al. [55]

To the best of our knowledge only two studies have been published describing vascular responses of isolated middle meningeal arteries from animals. In the first study, administration of PACAP1–38, PACAP1–27, and VIP to pre-contracted rat arterial segments did not cause any significant effect. Confirming the viability of the preparation, treatment with CGRP of the same arterial segments caused a 100% relaxation of the pre-contraction [34]. In the second study, rat middle meningeal arteries were mounted in a pressurized myograph system. In concentrations as low as 1–1000 pM, PACAP1–38 caused dilation of middle meningeal arteries that were blocked by the PAC1 receptor antagonist PACAP6–38 [55] (Fig. 3). It was suggested that PACAP1–38 affected middle meningeal artery tone by acting on a combination of two splice variants of the PAC1 receptor, namely the PAC1null and PAC1Hop1 receptor isoforms. Stimulation of PAC1 receptor causes in turn activation of the cyclic adenosine monophosphate/protein kinase A pathway leading to the opening of adenosine triphosphate sensitive potassium channels [56].

In man, PACAP1–38 and VIP induced only a weak relaxation of isolated middle meningeal arteries [31, 57]. VIP had a somewhat more potent effect on dilation than PACAP1–38. Neither the PAC1 antagonist PACAP6–38 nor the VPAC1 antagonist PG97–269 were able to block the PACAP1–38 induced relaxation suggesting the effect to be mediated via VPAC2 receptors [31].

#### In vivo

The genuine closed cranial window model has been used to study the effect of PACAP1–38, PACAP1–27, and VIP on rat middle meningeal artery *in vivo*. When given as a bolus i.v. infusion to anaesthetized rats a maximal dilation of ~60% was found for VIP and PACAP1–27, while the efficacy of PACAP1–38 was somewhat lower with a dilation of ~45%. Interestingly, the pD2 value of ~6 (in g/kg) for PACAP1–38 indicated a higher sensitivity of the middle meningeal artery as compared to PACAP1–27 with a pD2 value of ~5.5 [33]. In the presence of VPAC1 receptor antagonist (PG97–269) the response to PACAP1–38 but not to VIP was significantly decreased [33]. When given alone PACAP6–38 induced a slight dilation, but no significant inhibition of PACAP1–38 induced dilation of middle meningeal arteries was observed [33]. All together suggesting PACAP1–38 induced dilation of middle meningeal artery to be mediated via VPAC1 receptors. In another study, PG97–269 did not inhibit VIP and PACAP1-38 induced dilation of middle meningeal arteries. On the other hand, the VPAC1/VPAC2 antagonist VIP6–28 significantly inhibited VIP and PACAP1–38 induced dilation, suggesting VPAC2 receptors to be responsible [58]. This is in support of the findings in human meningeal arteries [31]. Thus, controversy exists whether VIP and PACAP induced dilation of rat meningeal arteries are mediated via VPAC1 or VPAC2 receptors.

**Fig. 4:**
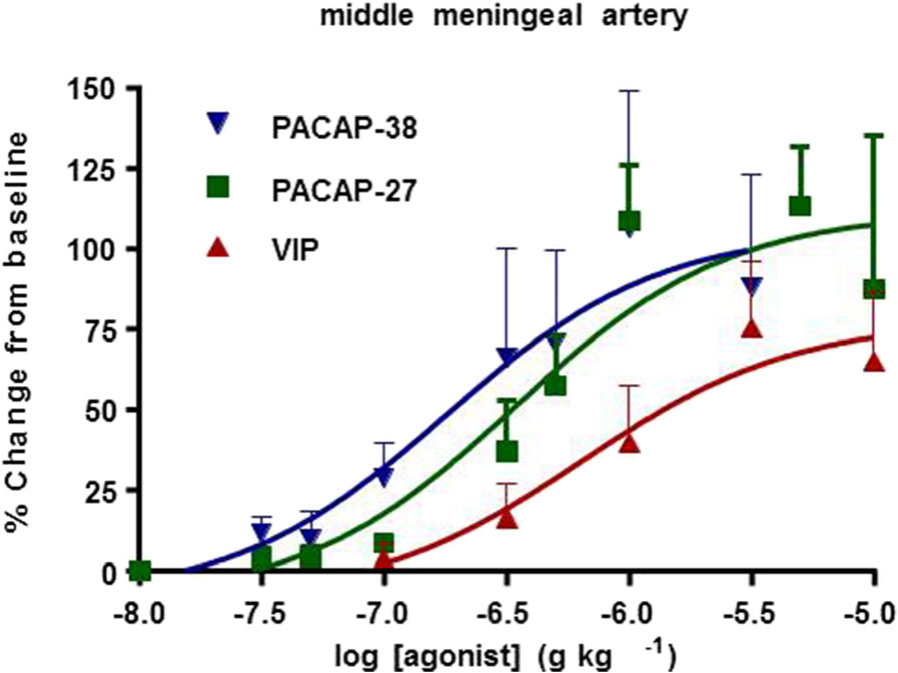
Effects of increasing doses (i.c.) of PACAP1–38, PACAP1–27 and VIP on middle meningeal artery diameter in the genuine closed cranial window model. Mean values ± SEM from 5 to 7 animals. Adapted from Bhatt et al. [59]

Intra carotid artery administration of PACAP1–38 induces an ED_50_ (the dose of a drug that gives half-maximal response) response in dural arteries at ten times lower concentrations of PACAP1–38 than after i.v. infusion [52]. Also, the maximum change in artery diameter from baseline was around 75% when given i.c. and 50% when given i.v. [52]. Increasing doses of PACAP1–38, PACAP1–27, and VIP administered as bolus i.c. infusion exhibited pD2-values of 6.7, 6.5, and 6.2, respectively. The maximum responses to PACAP1–38 and PACAP1–27 were around 105% (change from baseline) and VIP around 75% (Fig. 4) [59]. Because of variations between animals no significant differences between PACAP1–38, PACAP1–27 and VIP responses were observed.

### Mast cells and migraine

#### Mast cells

**Fig. 5:**
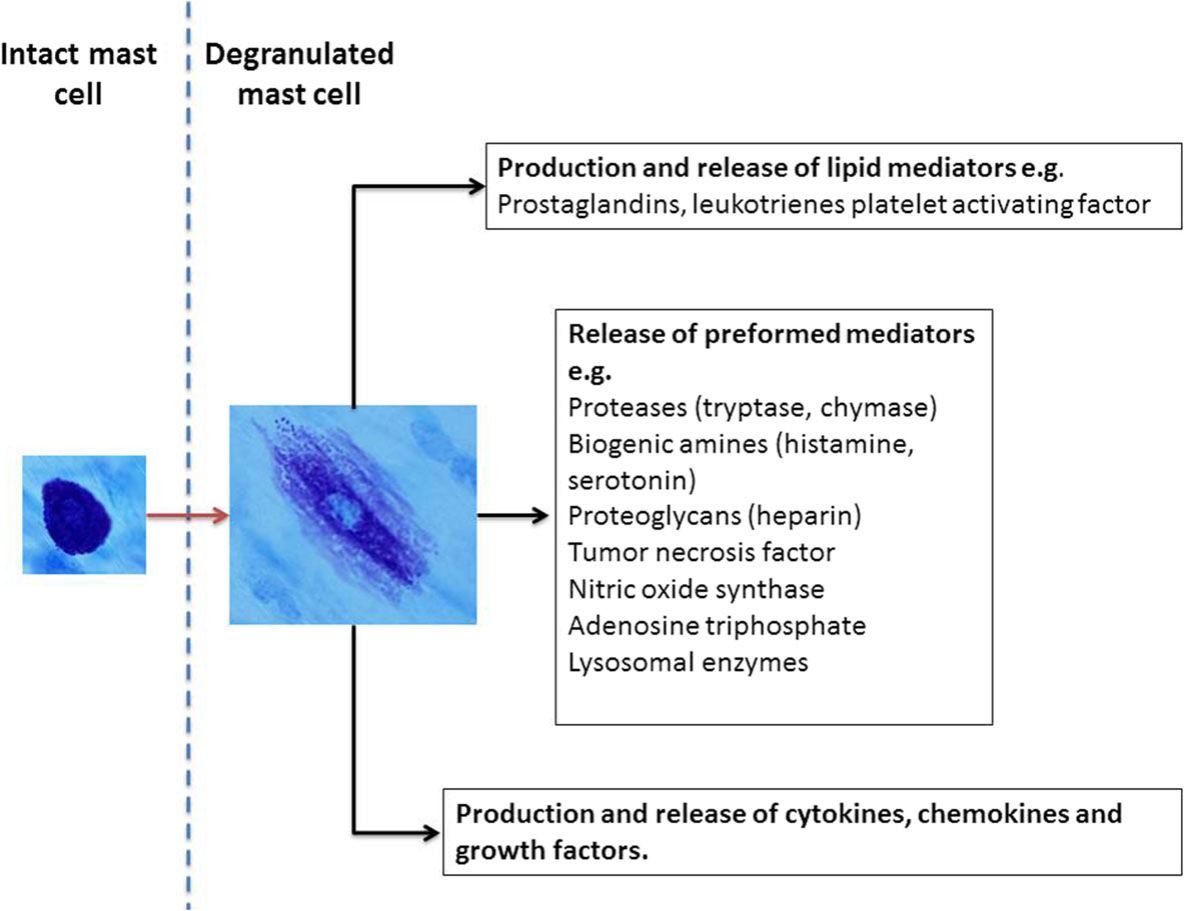
Toluidine blue stained intact and degranulated mast cell are shown together with a list of mast cell mediators [91]

Mast cells were first described in the late nineteenth century, but it was not until the 1950’s that part of their biological contribution to inflammatory allergic diseases became known through the discovery of histamine release. Mast cells contain vesicles comprising numerous inflammatory and vasodilatory substances (Fig. 5) and undergo degranulation upon activation by exogenous allergens or endogenous stimuli [60]. Mast cells are derived from pluripotent hematopoietic CD34+ stem cells in the bone marrow and circulate in the blood as progenitors before they acquire a mature phenotype in the microenvironment of their target tissue [61]. They are embedded in various tissues throughout the body and derive into either of two subtypes as referred to as mucosal or connective tissue type mast cells. The local cytokine environment conditions their subtype, but they hold an ability to adapt and change phenotype if needed [62, 63]. Mast cells embedded in skin, peritoneum, and dura mater are all of the connective tissue type, and thus peritoneal mast cells can potentially be used as a model for dura mater mast cells [64].

Two different signaling pathways leading to degranulation have been identified, namely the antigen and the basic secretagogue. The antigen pathway comprises stimulation through cross-linking with the high-affinity immunoglobulin E (IgE) receptors, FcεRI, and mast cells release their mediators to the local environment. Basic secretagogues stimulate mast cells to degranulate via G protein-dependent activation of phospholipase C. However, they can also be stimulated to degranulate via mechanical, thermal, or even receptor-independent mechanisms [65].

#### Clinical implications of mast cell-involvement in migraine

A correlation between mast cell function and migraine has been clinically implicated by significantly elevated plasma histamine levels in migraine patients, both during attacks and in interictal periods [66, 67]. For migraineurs there is a high comorbidity to histamine-driven conditions like allergic rhinitis, asthma, and food allergy [68–71] as compared to the general population [72]. Histamine-infusion to migraineurs induced an immediate headache during infusion, followed by a genuine migraine attack several hours later. This can be abolished by pretreatment with the histamine-receptor 1 (H1) antagonist, mepyramine [73]. However, histamine release alone is not responsible for spontaneous migraine attacks, as histamine-receptor H1 and H2 blockade is a poor prophylaxis for migraine sufferers [73, 74], indicating a discrepancy between genuine migraine attacks as compared to histamine-provoked attacks. Stimulation of histamine H3 receptors have been suggested to be involved in a negative feedback loop causing inhibition of histamine release from mast cells and C-fiber nerve endings [75]. The histamine catabolite N^α^-methylhistamine, that is about 3 times more active as an agonist on the H3 receptor, was found to be significantly better than placebo after prophylactic treatment twice a week for 12 weeks [75]. These findings are somewhat surprising, considering that H1- and H2 receptor antihistamines have not been effective in treating migraine [76].

In addition to histamine, mast cells release several chemical mediators such as prostaglandin I_2_ (PGI_2_), that has been shown to cause activation and sensitization of meningeal sensory afferents [77, 78] and to induce immediate headache in migraine patients and non-migraineurs as well as migraine-like attacks in migraineurs [43, 79]. Glyceryl trinitrate (GTN) is a potent migraine provoking substance that in low doses causes degranulation of dural mast cells after i.v. infusion to awake as well as anaesthetized rats [80, 81]. PACAP, but not VIP, has been shown to induce migraine headache as well as mast cell degranulation [10, 11, 82]. Thus, given their pro-inflammatory properties and their dense population in dura mater, mast cells are suggested to be involved in the pathophysiological processes leading to migraine [83–85].

### Characterization of PACAP-induced mast cell degranulation

**Fig. 6:**
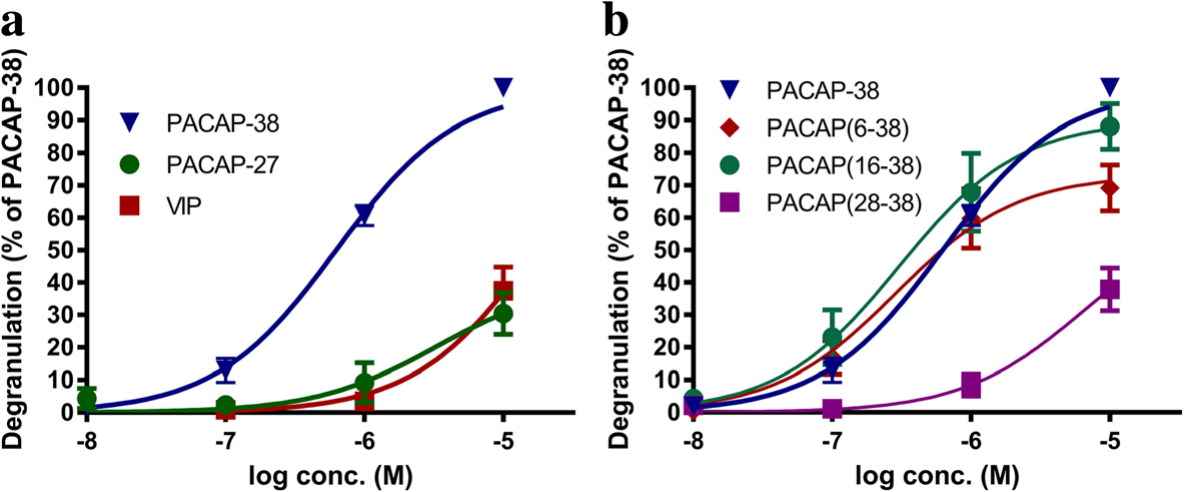
Degranulation of rat peritoneal mast cells expressed as percentage of PACAP1–38, which is the strongest mast cell degranulator tested. **a** Shows the effect of the endogenous peptides PACAP1–38, PACAP1–27, and VIP. **b** Shows the effect of PACAP1–38 and the fragments PACAP6–38, PACAP16–38, and PACAP28–38. Values are given as means ± SEM of 4–8 experiments. From Baun et al. [82]

**Fig. 7:**
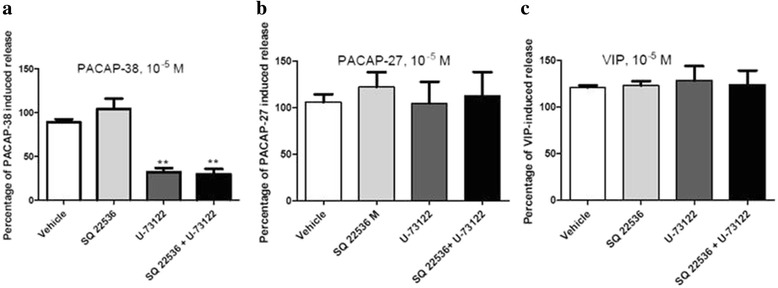
Degranulation of peritoneal mast cells induced by **a** PACAP1–38, **b** PACAP1–27 and **c** VIP in the presence of the adenylyl cyclase inhibitor SQ 22536 and the phospholipase C inhibitor U-73122 alone or in combination. Values are presented as amount of degranulation expressed as percentage of degranulation with each peptide alone. Values are given as mean ± SEM, *n* = 5; **p < 0.01 Mann Whitney U-test as compared to the vehicle group [82]

The mast cell degranulating effect of PACAP was first shown in human skin biopsies [86]. Single challenges with PACAP1–38, PACAP1–27, and VIP caused significant release of histamine peaking at 4 min after skin challenge. The release of histamine was significantly higher for VIP and PACAP1–27 as compared to PACAP1–38 [86]. In mice an intradermal injection of PACAP1–38 induced oedema and significant degranulation of mast cells [87]. In a more detailed study, mast cell degranulation induced by PACAP analogues, including both PAC1 receptor agonists and antagonists, was characterized in isolated rat peritoneal mast cells. PACAP1–38, PACAP1–27, VIP, PACAP6–38, PACAP16–38, and PACAP28–38 induced a concentration-dependent degranulation of the mast cells (Fig. 6). The compounds tested divided in two distinct groups, the efficient degranulators being PACAP1–38, PACAP6–38, and PACAP16–38 with pEC_50_ values between 6.6 and 6.2; interestingly, the PAC1 receptor antagonist PACAP6–38 is a member of this group. The other group consisted of weaker degranulators being PACAP1–27, VIP, and PACAP28–38 with pEC_50_ values between 5.5 and 4.8. Furthermore both, the PAC1 receptor agonist maxadilan, a 61–amino acid peptide with no significant sequence homology to PACAP [23], and the PAC1 receptor selective antagonist max.d.4, a modified fragment of maxadilan, showed no mast cell degranulating effects when applied at a concentration of up to 10^−5^ M [82]. These findings all suggest a PAC1 receptor independent mast cell degranulation and are further supported by a still unpublished study from our group where the PAC1 receptor antagonist M65 (another modified fragment of Maxadilan) failed to inhibit PACAP1–38 induced mast cell degranulation. Inhibition of intracellular mechanisms demonstrated that the phospholipase C inhibitor U-73122 significantly inhibited PACAP1–38- but not PACAP1–27- and VIP-induced mast cell degranulation (Fig. 7). The adenylyl cyclase inhibitor SQ 22536 has no effect on mast cell degranulation induced by either of the peptides. When taken together, the difference in potency between mast cell degranulating effects of PACAP1–38 and PACAP1–27 known to be equipotent on PAC1 receptors, the potent mast cell degranulating properties of the PAC1 receptor antagonist PACAP6–38 and the lack of inhibitory effect of M65 on PACAP1–38 induced mast cell degranulation, all suggest that degranulation is not mediated via the PAC1 receptor in rat [82].

### The role of PACAP1–38 induced mast cell degranulation on dural artery dilation

**Fig. 8:**
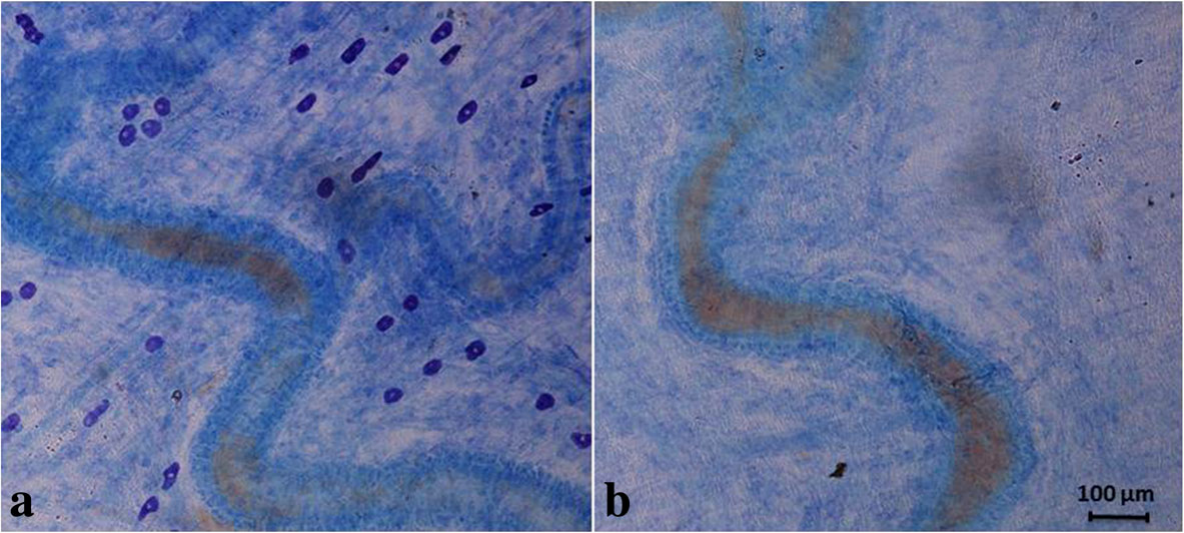
Toluidine blue staining revealed the presence of intact mast cells in dura mater from control rats (**a**) and the depletion of mast cells in dura mater from compound 48/80 treated rats (**b**)

**Fig. 9:**
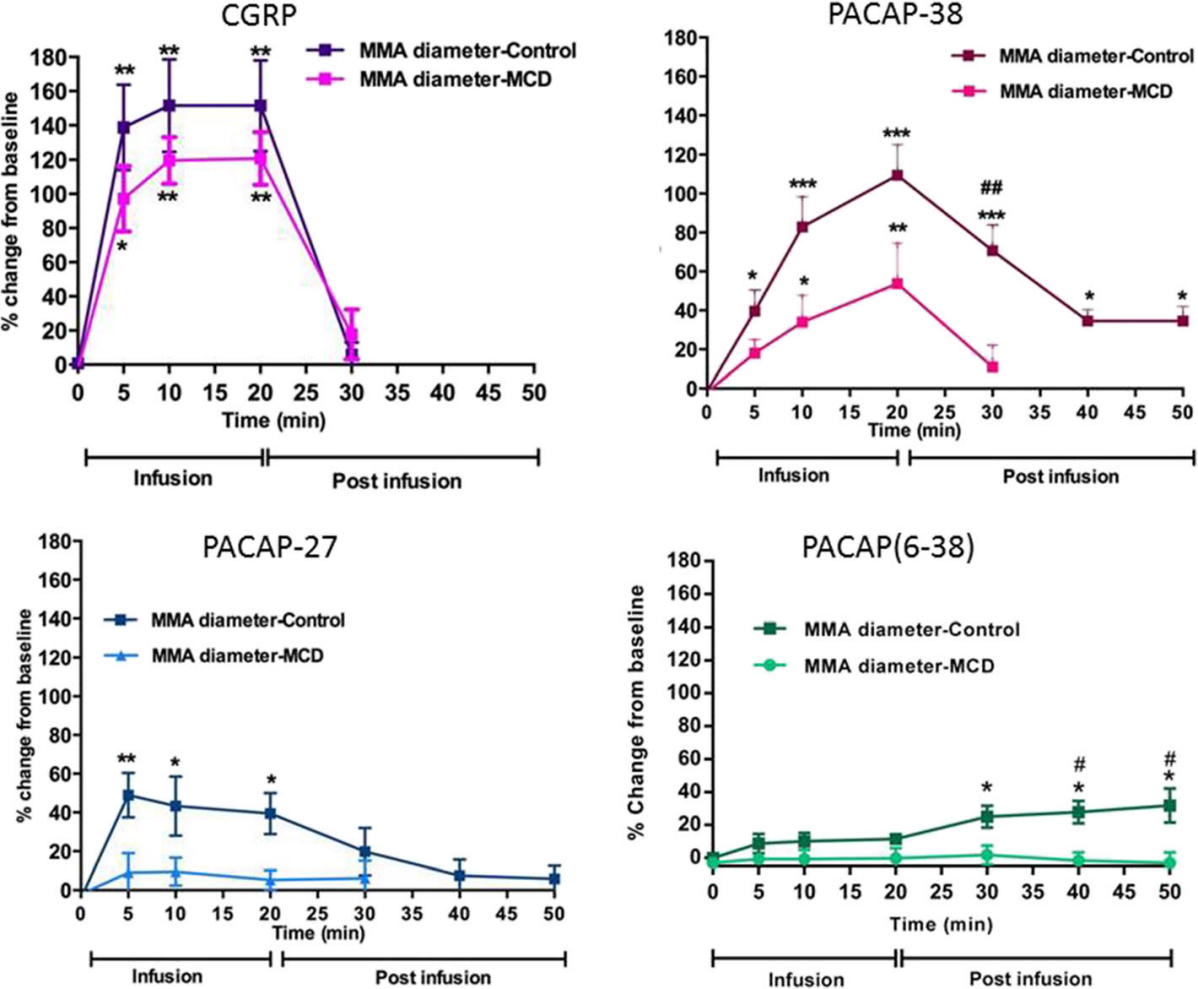
Middle meningeal artery (MMA) response to 20 min i.v. infusion of CGRP (0.25 μg kg^−1^ min^−1^), PACAP1–38 (0.4 μg kg^−1^ min^−1^), PACAP1–27 (0.4 μg kg^−1^ min^−1^) and PACAP6–38 (0.4 μg kg^−1^ min^−1^). The darker color represents experiments performed on control rats while experiments represented with the lighter color are performed in mast cell depleted (MCD) rats. Mean values ± SEM are given as percentage increase in MMA diameter from the pre-stimulation baseline. Statistical analysis by ANOVA (Kruskal-Wallis test) followed by Dunn’s comparison test to compare differences from baseline values (0) ***p < 0.001; **p < 0.01; *p < 0.5. ## *p* < 0.01; # *p* < 0.05 compared to the corresponding time point in MCD rats [59]

In healthy human volunteers PACAP1–38 was given as a 20 min infusion leading to vasodilation of the middle meningeal artery for up to five hours after infusion [10]. PACAP1–38 has an elimination half-life of 3.5 to 10 min [53, 88], hence the delayed effect cannot be attributed to a direct vascular effect of PACAP1–38, but rather to a cascade of events triggered by PACAP1–38. The strong degranulatory effect of PACAP1–38 on rat mast cells [82] and the dense population of mast cells found in apposition to dural arteries (Fig. 8) inspired our group to perform a set of experiments investigating the role of mast cell degranulation in middle meningeal artery dilation using the rat closed cranial window model. In these experiments one group of rats received repeated treatment with the secretagogue Compound 48/80, while the other group received vehicle. At the time of experiment, 4–5 days after the treatment, the mast cells were depleted of their granules (Fig. 8) [59]. In control rats a 20 min infusion of PACAP1–38, PACAP1–27, and CGRP but not VIP caused a significant increase in middle meningeal artery diameter. The response to CGRP returned to normal within 10 min after the end of infusion, while vasodilation induced by PACAP1–38 and PACAP1–27 showed a slower recovery. Fifty minutes after PACAP1–38 infusion, but not after PACAP1–27 infusion, the middle meningeal artery was still significantly dilated (Fig. 9) [59].

The PAC1 receptor antagonist PACAP6–38 exhibits potent mast cell degranulating properties [82], but without direct vascular effects. When infused over 20 min no significant change in middle meningeal artery diameter is observed. However, after termination of the infusion the artery starts to dilate and dilation reaches significance at 30 min, lasting until the end of experiment 50 min after the infusion. Chronic depletion of mast cells attenuates the responses to PACAP1–38 and PACAP1–27 and abolishes the delayed PACAP6–38 induced dilation (Fig. 9) [59]. This suggests that PACAP1–38 causes dilation of middle meningeal arteries partly due to mast cell degranulation. These effects might be responsible for long-lasting flushing and delayed migraine attacks observed after PACAP1–38 infusion.

Taking the results of all the described studies together, it is interesting to note that the PAC1 receptor antagonist PACAP6–38 is as potent a mast cell degranulator as PACAP1–38 and that the effect seems to be mediated via a non-PAC1 receptor. Furthermore, the weak mast cell degranulating effects of VIP suggest that VPAC1 and VPAC2 receptors are not involved. Though, PACAP6–38 is widely used as a PAC1 receptor antagonist it should be kept in mind that it has agonistic mast cell degranulating properties similar to that of PACAP1–38 [82] and thus hypothetically PACAP6–38 might cause hypersensitivity via this mechanism. The PAC1 receptor antagonists M65 and max.d.4 don’t share the mast cell depleting properties of PACAP6–38 and therefore should be preferred in studies characterizing the effects of PACAP on durally evoked hypersensitivity. The stimulatory effect of PACAP6–38 on a non-PAC1 receptor is supported by a study performed in a primary culture of trigeminal ganglion neurons from rat and mice in which, PACAP6–38, act as an agonist [89]. However, in this study the antagonists M65 (PAC1) and VIP6–28 (VPAC1 and VPAC2) share the agonistic features with PACAP6–38. The mast cell degranulation and migraine provoking effects of PACAP6–38 have not been investigated in humans. Presuming that the rank order of potency for these compounds to induce mast cell degranulation in humans equals that in rats, such a study would disclose if PACAP1–38 and PACAP6–38 have the same order of potency in headache provocation and if mast cell degranulation is involved in migraine pathophysiology. Such a study would also reveal if PACAP provoked migraine is induced by PAC1 receptors or via a yet unknown PACAP receptor.

## Conclusion

The few studies involving pharmacological characterization of PACAP- and VIP-induced relaxant responses of cerebral arteries from animals suggest the involvement of VPAC1 receptors. The mechanism for PACAP1–38 to cross the blood–brain barrier seems to be insufficient for transporting PACAP into the smooth muscle layer of the cerebral arteries in concentrations high enough to induce vasodilation after i.v. infusion of PACAP1–38.

Though isolated rat dural arteries do not respond to PACAP or VIP in a wire myograph system, PACAP1–27, PACAP1–38, and VIP show equipotent effects in studies performed on human middle meningeal arteries *in vitro* and rat dural arteries in vivo. In man, blockade experiments with VPAC1 and PAC1 receptor antagonists, suggests the dilation to be mediated via VPAC2 receptors. However, this assumption has not been confirmed by the use of selective antagonists for VPAC2 receptors. In rat, controversy exists weather VPAC1 or VPAC2 receptors are involved in PACAP1–38 induced meningeal artery vasodilation. As the PAC1 receptor has been suggested to be responsible for PACAP1–38 induced headache/migraine, the above described findings suggest PACAP1–38 induced headache/migraine not to be mediated via vascular responses. However, the extremely potent PAC1 receptor mediated effect of PACAP1–38 on middle meningeal arteries in a pressurized myograph system suggests a mechanism that can be involved in migraine pathophysiology. This finding was however, not observed *in vivo* after bolus or long term infusion with PACAP1–38 to rat or in vitro in wire myograph studies of human middle meningeal arteries.

Neurogenic inflammation involving degranulation of dural mast cells has been proposed to be part of the pathophysiological mechanisms of migraine. In rat, PACAP induces degranulation of peritoneal and dural mast cells via receptors coupled to phospholipase C. Long-term PACAP infusion causes middle meningeal artery dilation that partly is caused by degranulation of dural mast cells. Characterization of the responses suggests that the effect on mast cells is mediated via non-VPAC and –PAC1 receptors. Identifying such a receptor and a subsequent development of substances with selective antagonistic/inhibitory effect on this receptor, will open doors for more detailed studies on the role of mast cells in migraine pathophysiology. Another question to be answered is whether it is the PAC1 receptor or a still not identified receptor(s) that is (are) responsible for migraines provoked by PACAP.

## Abbreviations

CGRP: Calcitonin gene-related peptide
ED50: The dose of a drug that gives half-maximal response
FcεRI: Immunoglobulin E (IgE) receptor
i.c.: Intra carotid artery
i.v.: Intravenous
mRNA: Messenger RNA
PAC1: Pituitary adenylate cyclase activating polypeptide type I receptor
PACAP: Pituitary adenylate cyclase activating polypeptide
pD2: Negative logarithm of the molar concentration producing the half maximum response
VIP: Vasoactive intestinal peptide
VPAC1: Vasoactive intestinal peptide (VIP) receptor type 1
VPAC2: Vasoactive intestinal peptide (VIP) receptor type 2
